# A phase II study of the vitamin D analogue Seocalcitol in patients with inoperable hepatocellular carcinoma

**DOI:** 10.1038/sj.bjc.6601104

**Published:** 2003-07-15

**Authors:** K Dalhoff, J Dancey, L Astrup, T Skovsgaard, K J Hamberg, F J Lofts, O Rosmorduc, S Erlinger, J Bach Hansen, W P Steward, T Skov, F Burcharth, T R J Evans

**Affiliations:** 1 Department of Clinical Pharmacology, Rigshospitalet, Denmark; 2 Department of Hepatology, Rigshospitalet, Denmark; 3 Princess Margaret Division, The Toronto Hospital, Toronto, Canada; 4 Department of Hepatology, Aarhus Kommunehospital, Aarhus, Denmark; 5 Department of Oncology, Herlev Hospital, Herlev, Denmark; 6 LEO Pharma A/S, Ballerup, Denmark; 7 Department of Medical Oncology, St George's Hospital, London, UK; 8 Hèpato-Gastro-Entérologie, Hôpital Saint-Antoine, Paris, France; 9 Hèpato-Gastro-Entérologie 2, Hôpital Boujon, Clichy, France; 10 Department of Medical Gastroenterology, Aalborg Hospital, Aalborg, Denmark; 11 Medical Oncology, Leicester Royal Infirmary, Leicester, UK; 12 Department of Gastrointestinal Surgery, Herlev Hospital, Herlev, Denmark; 13 Department of Medical Oncology, University of Glasgow, UK

## Abstract

Hepatocellular carcinoma (HCC) is a common malignant tumour, which has a poor prognosis. Surgical resection can be curative but most patients are inoperable and most chemotherapy agents have minimal activity in this disease. Seocalcitol, a vitamin D analogue, induces differentiation and inhibits growth in cancer cell lines and *in vivo*. The vitamin D receptor is expressed in hepatocytes and more abundantly in HCC cells. In total, 56 patients with inoperable advanced HCC were included in an uncontrolled study of oral Seocalcitol treatment for up to 1 year (with possible extension for responders). The dose was titrated according to serum calcium levels. The treatment effect was evaluated by regular CT scans. Out of 33 patients evaluable for tumour response, two had complete response (CR), 12 stable disease and 19 progressive disease. The CRs appeared after 6 and 24 months of treatment, and lasted for 29 and at least 36 months (patient still in remission when data censored). Seocalcitol was well tolerated; the most frequent toxicity was hypercalcaemia and related symptoms. Most patients tolerated a daily dose of 10 *μ*g of Seocalcitol. This is the first study showing activity, by reduction in tumour dimensions, of a differentiating agent in patients with an advanced bulky, solid tumour. Seocalcitol may have an effect in the treatment of HCC, especially in early disease when a prolonged treatment can be instituted. The survival benefit with or without tumour response should be determined in controlled studies.

Hepatocellular carcinoma (HCC) is one of the most common malignant tumours in the world accounting for more than 300 000 cases per year (Parkin *et al*, 1993). There is a large geographical variation, with the annual incidence ranging from less than five cases per 100 000 persons in Europe and North America to around 20 per 100 000 in eastern Asia (Parkin *et al*, 1993). HCC is twice as common in males as in females, and a strong and consistent association between HCC and cirrhosis of the liver is found, which is independent of ethnic group and aetiology of the cirrhosis (HBV or HCV infection or alcohol) (Zaman *et al*, 1985). Surgical resection is considered the most effective treatment, but can rarely be performed and carries a high operative risk due to the poor general status of the patients and/or the reduced functional capacity of the residual cirrhotic liver. Furthermore, the cancer recurrence rate is approximately 50% at 1 year after potentially curative resection (Nagasue *et al*, 1990; Iwatsuki *et al*, 1991). Percutaneous ethanol injection, radiofrequency, and transarterial chemoembolisation are invasive techniques that have shown some efficacy in reducing tumour bulk, but their ability to improve survival has yet to be established (Yamada *et al*, 1990). Similarly, systemic chemotherapy may induce tumour responses, but survival benefit has not been clearly demonstrated, and the results of most chemotherapy agents are disappointing (Falkson *et al*, 1984; Bruix *et al*, 2001). In addition, the lack of efficacy of antiandrogens (Grimaldi *et al*, 1998) and tamoxifen (Gallo *et al*, 1998) has also been clearly demonstrated in unresectable HCC. Consequently, novel approaches are required for the management of inoperable HCC.

1*α*,25-dihydroxyvitamin D_3_ has been shown to exert potent cell regulatory effects in cells other than those involved in calcium homeostasis. The effects are thought to be mediated through binding to an intracellular receptor protein, the vitamin D receptor (VDR). The receptor–ligand complex interacts with DNA sequences, leading to activation or suppression of gene transcription. More than 50 genes have been shown to be regulated by this mechanism, among these the cell cycle regulators p21, p27, c-*fos* and c-*myc*. Regulation of these genes may in turn explain the antiproliferative and differentiating effects of vitamin D and analogues (for a review see (Hansen *et al* (2000)), as well as their ability to inhibit the invasive potential of human cancer cells *in vitro* (Hansen *et al*, 1994) and to inhibit tumour-induced angiogenesis (Majewski *et al*, 1993).

In search of a compound with less effect on calcium metabolism a new vitamin D analogue, Seocalcitol, has been synthesised (Hansen *et al*, 2000). Seocalcitol is 50–200 times more potent in inhibiting proliferation and inducing differentiation of human cancer cell lines than 1α,25-dihydroxyvitamin D_3_ (Hansen and Mäenpää, 1997). At the same time, Seocalcitol has 50% less calcaemic effect in rats relative to 1α,25-dihydroxyvitamin D_3_ (Mathiasen *et al*, 1993).

In tumour-bearing animals, Seocalcitol has been shown to suppress growth of chemically induced breast cancer (James *et al*, 1998) and xenografts of colon cancer (Akhter *et al*, 1997), breast cancer (Colston *et al*, 1992) and pancreatic cancer (Colston *et al*, 1997a), and to inhibit metastasis from prostate cancer (Lokeshwar *et al*, 1999). Vitamin D receptors have been reported to be present in the liver including human hepatocytes (Berger *et al*, 1988). Moreover, the expression of VDRs may be more pronounced in HCC tissue than in normal or cirrhotic liver tissue (Sakai *et al*, 1988). A study investigating the antiproliferative effects of 1α,25-dihydroxyvitamin D_3_ in seven liver cancer cell lines demonstrated that VDRs were present in all cell lines (Sakai *et al*, 1989). Seocalcitol can also profoundly inhibit the proliferation of the HCC cell line HEP-G2 compared to untreated controls (Akhter *et al*, 2001).

Seocalcitol was well tolerated in a phase I clinical study in patients with breast or colorectal cancer, and on the basis of this study the recommended daily dose for prolonged treatment was 7 *μ*g m^−2^ day^−1^ (Gulliford *et al*, 1998). Consequently, in view of the evidence supporting the expression of VDRs in hepatocellular cancers, and the lack of efficacious systemic therapies in this disease, a phase II study was performed in patients with inoperable hepatocellular cancer to determine the objective antitumour activity of Seocalcitol in this disease.

## MATERIALS AND METHODS

### Patients

The study was a multicentre, open, noncontrolled trial at centres in Denmark, France, UK, and Canada. The study was approved by the Research Ethics Committees or Institutional Review Boards of all participating institutions, and all patients gave written, informed consent.

Eligible patients were those with a diagnosis of HCC, verified by histology or cytology, who were not candidates for liver resection or liver transplantation, with at least one bidimensionally measurable lesion, age ⩾18 years, WHO performance status of 0–2, a life expectancy of at least 3 months, an albumin-corrected serum calcium of <2.65 mmol l^−1^, and adequate renal (serum creatinine ⩽2 times the upper limit of normal) and haematological (Hb ⩾10 g dl^−1^, WBC ⩾3.0 × 10^9^ l^−1^, platelets ⩾100 × 10^9^ l^−1^) function. Patients with a history of hypercalcaemia, disordered calcium metabolism, anticancer therapy within the previous 4 weeks, or calcium-lowering therapy within the previous 2 weeks, were excluded.

### Seocalcitol treatment schedules

Seocalcitol was administered once daily prior to the evening meal. A starting dose of 10 *μ*g day^−1^ was used in all patients. During an initial dose-finding phase, the individual patients dosage was increased every 2 weeks until a dose was reached that resulted in hypercalcaemia. Hypercalcaemia was initially defined as ionised serum calcium ⩾1.50 mmol l^−1^ or albumin-corrected serum calcium ⩾3.00 mmol l^−1^; later the limits were lowered to 1.40 and 2.80 mmol l^−1^, respectively because of clinical experience from this, and concomitant phase II studies, that marked hypercalcaemia was inevitable if the dose was further increased after the first limits were exceeded.

At ionised serum calcium ⩾1.50 mmol l^−1^ (albumin-corrected calcium ⩾3.00 mmol l^−1^) Seocalcitol was stopped for 1 week and then recommenced at the dose level immediately below the dose causing hypercalcaemia, and this was used as the maintenance dose unless further dose reduction was necessary because of hypercalcaemia. Dose levels of 5, 10, 15, 20, 30, 40, and 60 *μ*g daily were allowed. Patients could continue treatment for up to 1 year but were withdrawn earlier if there was evidence of disease progression or unacceptable toxicity. Treatment could be extended for patients having response or stable disease (SD) after 1 year.

Before starting Seocalcitol therapy, all patients were seen by a dietitian and the dietary calcium intake assessed. All patients were given advice on dietary calcium uptake and encouraged to adhere to a ‘lowest acceptable calcium diet’ (400 mg day^–1^ of calcium). Dietary compliance was checked at follow-up visits by individual investigators.

### Assessment of toxicity and antitumour effect

Prior to starting therapy, all patients underwent clinical assessment and measurement of full blood count, serum urea, electrolytes, liver function tests, glucose and alfa foeto-protein (AFP). Serum albumin, creatinine, total calcium and ionised calcium were also measured and the serum calcium corrected for albumin using the following formula: albumin-corrected serum calcium=total serum calcium+[(40–serum albumin) × 0.02]. Assessment of site, size, and spread of the HCC was performed by CT scan of the abdomen (and other sites as appropriate) and chest X-ray up to 2 weeks prior to starting therapy, and marker lesions with a diameter ⩾10 mm were identified.

The serum albumin, calcium (total), creatinine, and ionised calcium were measured weekly until the serum albumin-corrected calcium had been stable for 4 weeks at the maximum intended Seocalcitol dose. Thereafter samples were measured at 4-weekly intervals. The full blood count and other biochemical analyses, including AFP, were measured at 4-weekly intervals throughout the study.

Toxicity was graded by the CTC criteria (Green and Weiss, 1992) and was recorded at 4-weekly intervals. Similarly, clinical assessment was performed at 4-weekly intervals throughout the study. Disease assessments by CT scan were performed after 12, 24, and 52 weeks of treatment, and response assessments determined using the SWOG criteria (Green and Weiss, 1992). Tumour responses were confirmed on a repeat scan performed at least 4 weeks later.

### Statistics

The number of patients for inclusion in this study was determined according to Gehan's two-stage design for estimating the response rate (Gehan, 1961). Thus the sample size calculation was based on the requirement of stopping the study at an early stage if the response rate is below 20%, and of estimating the response rate with a standard error less than 0.10. Therefore if there was no objective response (complete or partial) among the first 14 patients, recruitment of patients would stop. If one or more responses were observed among these first 14 patients, up to 11 additional patients would be recruited.

Patients who did not complete at least 8 weeks of Seocalcitol at the maintenance dose were to be replaced. However, 21 patients were off study before 8 weeks of treatment (Table 3

**Table 3:** Treatment duration in 56 patients with advanced hepatocellular carcinoma included in the phase II trial with Seocalcitol

**Treatment duration with Seocalcitol (weeks)**	**No. of patients (%)**
<8	21 (37.5)
8–12	12 (21.4)
13–25	12 (21.4)
26–148	11 (19.6)

). Most of the 35 patients receiving treatment for at least 8 weeks had one or more dose reductions because of hypercalcaemia, and only 12 patients received treatment for at least 8 weeks at the individual maintenance dose. Therefore, it was decided that the formal stopping rule of Gehan's design could not be applied, and the enrolment was stopped by an *ad hoc* decision.

The time to disease progression was defined as the time from start of treatment until progression was first detected or the patient went off study due to clinical deterioration (without tumour measurements) or the need for other anticancer treatment. Overall survival was measured from the start of Seocalcitol treatment until death from any cause, and analyses performed using the Kaplan–Meier method.

## RESULTS

### Patient characteristics

A total of 56 patients were enrolled over a 2-year period from November 1996 from 12 centres in Denmark, France, UK, and Canada. Of these, four did not fulfil all in- and exclusion criteria and two died during the period between the screening visit and the start of treatment visit. Histological subtype was available for 10 patients, four of whom had fibrolamellar HCC. In total 50 patients received Seocalcitol treatment and were evaluable for assessment of safety. In total, 33 patients had repeat disease assessments after 12 weeks therapy and were evaluable for response. The remaining 23 patients died or had clinical disease progression before the first follow-up CT-assessment, and objective tumour response could therefore not be assessed (Table 1

**Table 1:** Baseline pretreatment characteristics for 56 patients included in the phase II trial of Seocalcitol for advanced hepatocellular carcinoma

**Characteristics**	**No. of patients (%)**
Gender	
Male	42 (75.0)
Female	14 (25.0)
	
Extent of disease	
Local	41 (73.2)
Metastatic	15 (26.8)
To lung/chest wall	8
To spleen, retroperitoneum, other abdomen	6
To bone and abdomen	1
	
Previous treatment^a^	
None	34
Surgery	14
Chemotherapy	8
Hormone/immunotherapy	4
	
Performance status (WHO)^b^	
0	26 (47.3)
1	22 (40.0)
2	7 (12.7)
	
Age (years), median (range)	62.5 (25–90)
	
AFP (ng ml^−1^), median (range)	297 (below detection limit–204,765,000)

a A patient can have more than one treatment.

b Missing observation for one patient.

).

The median age was 62.5 years (range 25–90), 42 (75%) were male, 14 were female, and 48 (87%) were of WHO performance status ⩽1, with 41 (73%) of patients having locally advanced disease only, and 15 (27%) having metastases. In total, 34 patients had received no other treatment before inclusion in the trial. Previous treatment (22 patients) included surgical resection (14), chemotherapy (eight, including alcohol injection in three) and hormone- or immunotherapy (4), with three patients having received more than one previous treatment modality.

### Efficacy analyses

The best responses of individual patients included two complete responses (CRs), 12 patients with SD, and 19 with progressive disease (PD) at 12 weeks (Table 2

**Table 2:** Outcome in 56 patients with advanced hepatocellular carcinoma included in the phase II trial with Seocalcitol

**Best response**	**No. of patients**
Complete response	2
Stable disease	12
Progressive disease	19
Not assessed	23
	
Time to progression median (range)	84.5 (0–833) days
	
Survival, median (range)	207 (0–1092^a^) days
	
1-year survival	28.6%
2-year survival	5.4%

a The last patient was censored at 1092 days.

), for an overall response rate of 3.5%. of the intention-to-treat population. The median time to progression was 84.5 days, and the median overall survival was 207 days (Table 2, Figure 1

**Figure 1:**
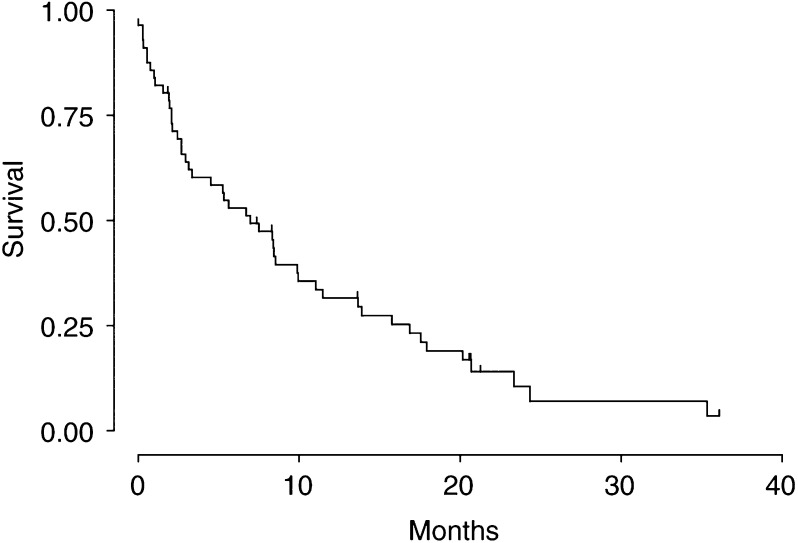
Survival of 56 patients with advanced hepatocellular carcinoma included in the trial phase II trial of Seocalcitol.

). Among the 12 patients with disease stabilisation as best response, the median time with SD was 101 days.

One of the patients with a CR was a 77-year-old man with cirrhosis of unknown aetiology. He had three tumours, the largest of which was 18 × 18 mm. Biopsy showed highly differentiated HCC. The CR was first documented after 24 weeks of treatment. After 44 weeks, there was a 10 × 10 mm relapse. The patient remained on treatment, and a second CR was obtained 22 weeks later. The patient was withdrawn from the study due to disease progression after 29 months. Alfa foeto-protein was less than 10 ng ml^–1^ for the first year, then gradually rose to around 330 ng ml^–1^. The Seocalcitol dose was between 10 and 20 *μ*g day^−1^ during the first 20 weeks, and thereafter maintained at 5 *μ*g day^–1^. Vitamin D receptors were present in this patient's tumour as determined by Western blot (data not shown).

The other patient who had a CR was a 43-year-old woman who had no history of cirrhosis, alcohol abuse or hepatitis virus infection. She had previously undergone a partial hepatectomy for a highly differentiated HCC. The patient had three recurrent tumours at inclusion into the study, the largest of which was 35 × 50 mm. Complete response was first observed after 103 weeks of treatment. Alfa foeto-protein was high initially, decreasing gradually during treatment, and eventually ended within normal limits. When last seen, the patient had been on treatment for 36 months and still had a CR. The dose was between 15 and 20 *μ*g day^–1^ during the first 50 weeks, thereafter 10 *μ*g day^–1^ (disregarding short treatment pauses because of hypercalcaemia). Tissue was not available to determine VDR expression in this patient's tumour.

### Calcium level and Seocalcitol dose

Table 3 gives Seocalcitol treatment duration in all treated patients. In total, 35 patients (69%) were treated for at least 8 weeks, and 11 for at least 26 weeks. Among the 35 patients who received treatment for at least 8 weeks, only 12 tolerated the dose that was determined in the dose-finding phase (the individual maximum tolerated dose) for at least 8 weeks. The remaining patients developed hypercalcaemia and had further dose reductions.

### Toxicity

The most frequent toxicity was dose-related hypercalcaemia which was observed in 80% of the patients (Table 4

**Table 4:** The most common (>4% of patients) adverse drug reactions

**Adverse drug reaction**	**Number of patients experiencing the adverse reaction**	**Percent of patients experiencing the adverse reaction**
Hypercalcaemia	40	80
Nausea	10	20
Malaise/fatigue/lethargy	5	10
Vomiting	5	10
Anorexia	3	6
Constipation	3	6
Creatinine	3	6
Dizziness/vertigo	3	6
Metabolic, other	3	6
Diarrhoea	2	4
Gastrointestinal, other	2	4
Salivary	2	4
Taste	2	4

). Hypercalcaemia usually resolved after 1 week without treatment. Other toxicities included nausea (20%), and fatigue (10%). In total, 81 of 96 reported reactions were CTC grades 1-2, 11 were CTC grade 3 (10 hypercalcaemia, one malaise/fatigue/lethargy), and four were CTC grade 4 (all hypercalcaemia).

## DISCUSSION

Seocalcitol is a differentiating agent which has shown promise in animal models of cancer (Akhter *et al*, 1997; Colston *et al*, 1997a; Lokeshwar *et al*, 1999). Previous studies with this vitamin D analogue in breast and colorectal cancer (Gulliford *et al*, 1998) and pancreatic cancer (Evans *et al*, 2002) have not demonstrated any evidence of antitumour activity as determined by objective reduction in tumour volume. At the time that this study was initiated, the primary end point was objective tumour response as defined by classical criteria based on anatomical changes in tumour dimensions. Consequently, with a response rate of 3.5%, the conclusion is that the agent is inactive in this disease using a response rate of 20% as the definition of activity in Gehan's design. However, Seocalcitol induced two CRs in patients with advanced HCC. Seocalcitol is likely to be cytostatic rather than cytotoxic, with disease stabilisation the likely clinical manifestation of any antitumour activity. Consequently the observation of two patients with CR raises the intriguing notion that Seocalcitol may have some antitumour activity in advanced HCC. Furthermore, the responses were durable, lasting for 29 months and at least 36 months. The rarity of spontaneous tumour regression in this disease, and the durability of the responses observed, support the notion that Seocalcitol may have activity in this disease.

Previous studies of differentiating agents in malignant disease have mainly focused on the use of retinoids as cancer chemoprevention agents (reviewed in (Evans and Kaye, 1999)), although all-*trans* retinoic acid as induction or maintenance treatment can improve disease-free and overall survival when given in combination with chemotherapy in APML (Tallman *et al*, 1997), and isotretinonin can improve event-free survival following myeloablative chemotherapy and autologous bone marrow transplantation in children with high-risk neuroblastoma (Matthay *et al*, 1999). In contrast to these studies of maintenance retinoids in combination with chemotherapy in minimal residual disease, the present study is the first human study in which a differentiating agent has shown activity in reducing tumour volume in solid tumours when given as a single agent in advanced, bulky disease, albeit in a small percentage of patients.

This observation requires further investigation within randomised controlled trials comparing Seocalcitol with ‘standard’ therapy, with overall survival as the primary end point, which may be a more appropriate measure of efficacy for a differentiating agent, which may inhibit tumour growth rate rather than reduce tumour size.

It may be speculated that the response rate could be increased by selecting patients whose tumour expresses the VDR. Information on individual patients' VDR status is, however, difficult to obtain in a clinical study setting, since reliable immunohistochemical methods for the determination of VDR (on archived, formalin-fixed biopsies) are not available. Attempts were made in the present study to obtain snap frozen biopsies from tumours for the determination of VDR by Western blot where possible, although this was not an entry criterion for the study. Most patients had already undergone a diagnostic biopsy before study entry and repeat biopsies could not be justified for study purposes alone on ethical grounds. Similarly, the lack of frozen tumour material also meant that determination of VDR mRNA expression was not possible in this study.

In human liver cancers, one study has found VDR mRNA expression in all 10 tumours tested (Miyaguchi and Watanabe, 2000), another study found VDR in 10 of 18 tumours tested with a radioreceptor assay (Sakai *et al*, 1988). Finally, *in vitro* studies indicate that the VDR may be induced by the treatment with vitamin D and analogues (Mangelsdorf *et al*, 1987; McDonell *et al*, 1987; Mahonen *et al*, 1991; Arbour *et al*, 1993; Jääskeläinen *et al*, 2000). Thus, whereas for example antioestrogen treatment relies to a large extent on the presence of the oestrogen receptor, the effect of Seocalcitol may not be dependent on the pretreatment presence of VDR. However, selecting patients in future studies in HCC on the basis of BCLC or Okuda staging may be more relevant in identifying patients with small volume, slow-growing disease that may respond to Seocalcitol, as was the case for the two patients who had CR in this study. Furthermore, a significant number of patients in this study had PD and discontinued Seocalcitol before receiving 8 weeks of therapy. The toxicity profile of Seocalcitol differs significantly from that of chemotherapy or trans-arterial chemo-embolisation (TACE). Consequently, future studies could explore the combination of Seocalcitol with TACE in comparison with TACE alone in selected patients with unresectable HCC to determine if Seocalcitol can confer any progression-free survival advantage when combined with this regional therapeutic approach, thereby also overcoming the drawback of early disease progression in patients treated with a putative cytostatic agent alone.

Dose-dependent hypercalcaemia was the most frequent side effect. This was expected since the design of the study entailed dose increments every 2 weeks until hypercalcaemia or near-hypercalcaemia. The incremental design was chosen because of evidence of dose-dependent response in *in vivo* studies (Colston *et al*, 1992; Colston *et al*, 1997b; James *et al*, 1998). No cases of renal stone were observed. A maintenance dose of 10 *μ*g day^−1^ was acceptable for most patients. Other frequent side effects were mild, and included nausea and fatigue, which were often interpreted as secondary to the hypercalcaemia, but may also have been attributable to the primary disease.

In conclusion, activity of Seocalcitol in HCC was demonstrated, although this was less than the 20% objective response that is indicative of activity within the Gehan design. The side effects of the treatment were minimal and associated with hypercalcaemia. Randomised controlled trials are necessary to assess the efficacy of Seocalcitol with regard to survival, and to identify patient characteristics that may predict benefit from the treatment. The use of Seocalcitol as an adjuvant therapy after potentially curative resection (ie in ‘minimal disease states’) in HCC is an intriguing notion.
